# Renouncing care in French Guiana: the national health barometer survey

**DOI:** 10.1186/s12913-019-3895-6

**Published:** 2019-02-06

**Authors:** Astrid Van Melle, Claire Cropet, Marie-Claire Parriault, Leila Adriouch, Hélène Lamaison, Francine Sasson, Hélène Duplan, Jean-Baptiste Richard, Mathieu Nacher

**Affiliations:** 1 Centre Hospitalier de Cayenne – Centre d’Investigation Clinique INSERM, CIE1424, 3 avenue des Flamboyants, BP6006, 97306 Cayenne, CEDEX Guyane France; 2 COREVIH Guyane, Centre Hospitalier de Cayenne, Cayenne, Guyane France; 3 Association Guyane Promo Santé – IREPS Guyane, Cayenne, France; 4 Agence Régionale de Santé de la Guyane, Cayenne, France; 5 Santé publique France, Saint-Maurice, France

**Keywords:** Renouncing care, Health inequalities, Health system use, French Guiana, Health insurance coverage

## Abstract

**Background:**

In French Guiana, health inequalities are patent for a broad range of pathologies for all age groups. The objective of the present study was to quantify the proportion of the population that had renounced care in the past year, to study predictive factors, and to compare results with other French territories.

**Methods:**

A two-stage random sample of 2015 individuals aged 15 to 75 years was surveyed by telephone. A descriptive analysis of variables relative to renouncing care, use of health care, screening, and vaccination was initially performed. Multivariate analysis was then used to determine variables associated with renouncing care for financial reasons and renouncing for reasons linked to time were directly estimated using a Poisson model on weighted data. Variables with a significance level < 0.2 in the bivariate analysis were included in the full multivariate model.

**Results:**

In French Guiana, during the past 12 months, 30.9% of surveyed persons renounced care whatever the type for financial reasons. Results of the multivariate analysis showed that gender, perceived financial situation, perceived health and complementary insurance status were independent predictive factors of care renouncement for financial reasons. Overall, 24% of the surveyed population declared having renounced to care for time-related motives. The independent predictors for time-related renouncing were different than those for renouncing care for financial reasons: a higher education level and a poor perceived health were independently associated with time-related renouncement; retired persons and students were found to renounce care less frequently than persons with a job.

**Conclusions:**

Renouncing for financial reasons, a major target of the 2016 health law, represented a public health problem in French Guiana. Renouncing for lack of time was an important motive for renouncing, which is aggravated by the insufficient number of health professionals, but may benefit from organizational solutions. There are avenues for improvement of health for the most vulnerable: promote health, act on risk factors, and facilitate the readability and accessibility of the health system. Recent reforms to stabilize health insurance may however have some adverse consequences for migrants.

## Background

Health inequalities remain salient between different countries and within a given country [1]. The absolute and relative wealth levels are important but relative differences are prominent [2, 3]. Income inequalities are compounded by health inequalities, with rich countries generally having better health indicators than poor countries [4]. However there is a great heterogeneity between situations [5]. Technical progress in medicine, quantitative or qualitative increases in health services do not necessarily translate in the reduction of health inequalities. Universal health care may contribute to the reduction of health inequalities, but not necessarily, the average health may improve without eliminating the social gradient. There may be several explanations for this: diverse population categories may, at a constant health level, have different preoccupations relative to health; there may be informational barriers that lead the poorest and least educated to have a different relation to health and their body, and to have less knowledge of the health care system. Hence, health-seeking behavior tends to be late and more oriented towards curative care in this sub-population; in addition, financial barriers (remaining health costs, transport…) may also explain the social gradient for health. The reasons why patients are tested late, and/or let serious diseases without treatment and care are complex. Certain persons do not seek care, in other words they do not use the available public health-services [6] but others will renounce care, which suggests that obstacles ultimately lead them to renounce health care despite recognizing a need. The difference between not using and renouncing may be fuzzy. Renouncing care can be classified in 3 causal categories [7]: acceptability, availability, and accessibility. Acceptability corresponds to a personal choice, leading persons to renounce care because it is personally or culturally not acceptable. Availability and accessibility directly pertain to the health system and are endured by the patient. The notion of availability concerns the presence of infrastructures and/or delays to access them. Accessibility concerns transport and cost. For accessibility and availability public policies have the power to improve things.

Studies of the use of health services may aim for immediate application in the formulation and implementation of public policy [8] but they may have the broader and more challenging aim to understand why services are used or not used [9]. Researchers have attempted to link different variables to the individual’s likelihood of perceiving an event as a symptom or to his mode of responding to a symptom. There is a social class gradient in terms of the likelihood of interpreting a particular sign as a symptom [10]. Ethnic values also affect the decision to seek medical attention and the differential interpretation of objectively similar symptoms [11, 12].

On the opposite end of this equation lies a highly variable and crucial element: behavior. Health behavior has been defined as “any activity undertaken by a person who believes himself to be healthy, for the purpose of preventing disease or detecting disease in an asymptomatic stage.” Illness behavior has been defined as “any activity undertaken by a person who feels ill, for the purpose of defining the state of his health and of discovering suitable remedy.” Finally, sick-role behavior “is the activity undertaken by those who consider themselves ill for the purpose of getting well” [13].

Health or illness behavior refer to the subjective world of the behaving individual and not with the “objective” world of medical science [13]. The two are only partly correlated. Thus, persons unconcerned with a certain aspect of their health will not be likely to perceive any health services or information pertaining to that aspect of their health. Without concern or motivation for action, the environment will be preferably perceived in accordance with the person’s motives [14]. Moreover, underlying emotional aspects have greater influence for behavior than cognitive aspects. Perceived susceptibility, perceived seriousness, and perceived benefits of taking action and barriers to taking action, and readiness to take action vary widely between individuals. Depending on the various levels of these elements, and the presence of sufficient cues to trigger action, individuals may or may not use health services [13, 15].

However, the above individual aspects of behavior should not occult the fundamental importance of the social context in decision processes. Xenophobia, lack of transport, for instance, may thus selectively hamper access to care of vulnerable populations. Hence, the perception of an inclusive social system (education, health…) or a hostile system may have far reaching consequences of behavior towards the system, even before persons become ill.

French Guiana is a French territory located between Brazil and Suriname. It has the administrative status of a French “Departement” and Region, as found in mainland France. Persons (French citizens or documented foreigners) have access to a universal health care system with health insurance, the “sécurité sociale”, for employed persons, for low resource persons, and undocumented foreigners who have been staying for over 3 months on the territory have access to health insurance (Aide Médicale Etat). The GDP per capita is the highest in Latin America, and French Guiana is thus attractive for populations in search for better economic opportunities. French Guiana is a multicultural territory with populations of African, Asian, or European ancestry. Regarding health care, the level of care is often much better than elsewhere in Latin America, with rights and public funding that are unmatched on the continent. Paradoxically, in comparison with other French territories, French Guiana faces numerous challenges [16]: demography, with the highest population growth rate on the continent, socio economic problems, with a high unemployment rate, with 30% of immigrants generally living in poverty and not speaking French, and frequent illiteracy [16, 17]. A large proportion of the population does not have a valid health insurance, which restricts their access to care. Hence, although the Emergency department is always accessible for acute problems, structures dealing with chronic conditions may be more complicated to access in the absence of health insurance.

In French Guiana, health inequalities are patent for a broad range of pathologies for all age groups, from infectious diseases to non-communicable diseases. Thus undocumented immigrants reported a worse health when compared to others immigrants, and reported health declined over time [18]; immigrants were also diagnosed later for human immunodeficiency virus (HIV) [19], they were more susceptible to interrupt HIV follow-up and treatment [20]; for cancer, immigrants had more advanced stages and a poorer prognosis for cervical cancer [21] and a much lower survival for breast cancer [22]. At the Emergency ward in Saint Laurent du Maroni, the severity and the hospitalization rate were higher among undocumented immigrants [23]. An epidemic of Beriberi was recently reported among illegal gold miners in French Guiana [24]. In all the above studies, being an immigrant is used as a proxy to poverty, vulnerability and difficulties in accessing care leading to patent health inequalities. A recent study in the poorest neighborhoods around Cayenne showed that although three quarters of this population (73.4%) declared that it was easy to see a physician, 21% had renounced care in French Guiana [25]. The main reasons identified by multivariate analysis were « shyness » to ask questions when interacting with the administration or health structures, having previously been denied care by a physician. On the contrary, having previously had a family doctor reduced the likelihood of renouncing care. Thus, for the most vulnerable, negative experiences seemed to be generalized and perceived as a sign of non-eligibility or exclusion. However, counter-intuitively, 66.4% of persons having renounced care said it was easy to see a doctor. This study was based on a convenience sample in the precarious areas and did not allow making inferences beyond the sampled population.

The Health barometer survey is conducted by Santé Publique France, the French National public health agency, among the general population living in metropolitan area. For the first time, in 2014, this survey was conducted in four overseas territories, including French Guiana. The objective of the present study was to quantify the proportion of the randomly selected population that had renounced care in the past year, to study predictive factors in this socially and culturally complex isolated territory, and to compare results with other French territories.

## Methods

The 2014 Health Barometer survey uses a telephone and computer assistance and the overseas survey relies on a method similar to that of the « Health Barometer » study conducted in mainland France [25]. Health Barometer survey uses a two-stage random sample: sampling telephone numbers and sampling a single respondent within the eligible persons using one telephone number. First, phone numbers, fixed and cellular, were randomly generated. Then one person was randomly selected among eligible persons living in the household. To be eligible, a household had to include at least one person aged 15 to 75 years, residing in French Guiana, speaking French or Creole. Anonymity and respect of confidentiality were guaranteed using a procedure erasing the phone number. This procedure was approved by the French regulatory authority, the Commission Nationale de l’Informatique et des Libertés (CNIL). The study was conducted between April and November 2014. The refusal rate seemed lower than in mainland France (9% vs 25%), but a greater proportion of numbers remained unreachable (39% vs 18%). The participation rate was thus 49, 3% of interviews were abandoned. The mean duration of the questionnaire administration was 37 min. Data from French Guiana were weighed using sampling weights, taking into account the probability of drawing the phone number, the number of eligible individuals, and of phone lines within the household, then calibrated with reference data from the French National Institute for Statistics (2011 French Guiana population census). This calibration takes into account gender, age, education level, and household structure. The sample consisted of 2015 individuals aged 15 to 75 years [26]. More details concerning the survey methodology of the Health Barometer have been published elsewhere [27].

All analyses were performed with Stata® 13.0 software(College Station, Texas), using survey commands.

A descriptive analysis of variables relative to renouncing care, use of health care, screening, and vaccination was initially performed. The test used for bivariate analyses was Pearson’s Chi^2^ for weighted data using the second order Rao-Scott correction.

Multivariate analysis was then used to determine which variables were associated with renouncing care for financial reasons and which variables were associated with renouncing for reasons linked to time. As the prevalence of the outcomes of interest were greater than 10%, the common approximation of prevalence ratios by odds ratios using a logistic model was not appropriate; this would have led to overstate the effects size. Thus, prevalence ratios were directly estimated using a Poisson model on weighted data. Variables with a significance level < 0.2 in the bivariate analysis were included in the full multivariate model. This broad threshold allowed taking into account variables for which measures of association were potentially confounded. Variance inflation factors (VIF) were studied to avoid collinearity between variables included in the full multivariate model: the VIF for all included variables were < 1.5.A backward regression method was then used to keep in the final multivariate model only factors significant at the 0.05 level. Interactions between explanatory variables were then tested and removed if non-significant. Complementary analyses were performed to interpret factors associated with renouncing care for reasons linked to time. Indeed, although there was no multicolinearity, there were relations between the predictor variables. The analysis of interaction terms showed several significant interactions complicating the interpretation of the model. Thus we performed a multiple correspondences analysis to characterize patient profiles associated with renouncing care for reasons linked to time.

## Results

### Use of the health system

**Fig. 1:**
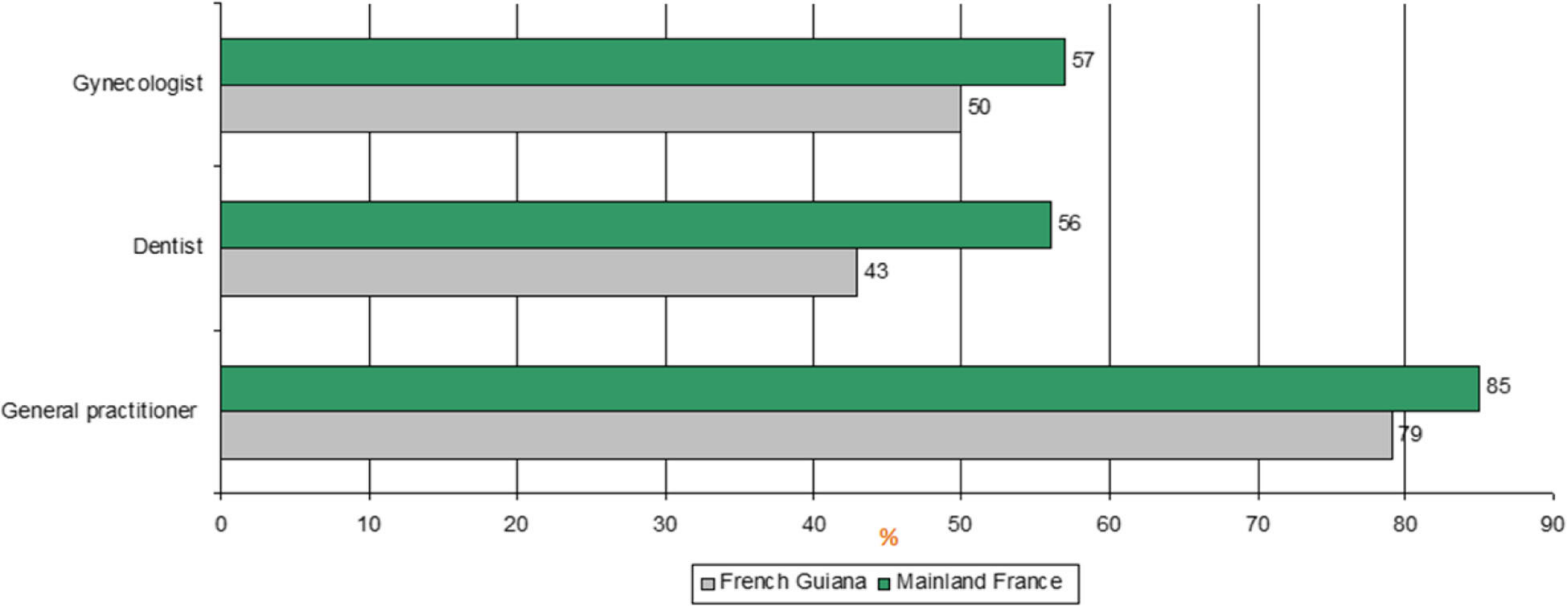
Use of health services in the 12 months prior to the survey in French Guiana and in Mainland France**.** Legend: Consultation in the 12 months prior to the survey

In 2014, 79% of persons had consulted a general practitioner for themselves at least once (71% of men, 85% of women), 43% had consulted a dentist at least once (36% of men, 49% of women); these levels were lower than those of other French territories (85% for general practitioners and 56% for dentists) (Fig. 1). Half of French Guianese women consulted a gynecologist in the past year, a proportion which was similar to other overseas territories but lower than in mainland France (*57%*) (Fig. 1). This difference was strongly linked to age: the frequencies of gynecologic consultations were comparable before 45 years (55% vs *60%*), but in women over 45 years, gynecologic consultations were much less frequent in French Guiana relative to mainland France (37% vs *54%*) [26].

When looking at the use of the health system in the subpopulation expected to require consultations by a health professional, diabetic persons (declaring having been told by their doctor to be diabetic) were less likely to have consulted a general practitioner or a specialist in the past 12 months relative to the French territory of La Réunion, the only territory with comparable data (Table 1).

**Table 1:** Use of health service in the 12 months prior to the survey among diabetic persons

Among diabetic persons		In the 12 months prior to the survey			
		Have consulted a general practitioner		Have consulted a specialist	
		Yes	Chi^2^	Yes	Chi^2^
French Guiana (*N* = 118)	%	83.2	*p* < 0.01	29.8	*p* < 0.05
La Réunion (*N* = 166)	%	95.4		45.8	
Total	%	91.7		39.7	
	N	263		121	

Similarly, persons declaring frequent or very frequent dental problems were less likely to have consulted a dentist in French Guiana than in other overseas French territories (Table 2).

**Table 2:** Use of health service in the 12 months prior to the survey among people who have dental problems or who have difficulty with their glasses or contact lenses

In the 12 months prior to the survey	Among people who often or very often have dental problems		Among people who have difficulty with their glasses or contact lenses	
	Have consulted a dentist (%)		Have consulted an ophtalmologist (%)	
	Yes	Chi^2^	Yes	Chi^2^
Martinique	70.1	*p* < 0.001	52.9	*p* = 0.47
Guadeloupe	64.3		52.3	
French Guiana	45.8		44.7	
La Réunion	71.7		55.1	
Total	%	60.8		50.9
	N	431		452

There was no significant difference regarding consultations of an ophtalmologist for persons declaring visual problems despite their glasses or contact lenses.

### Renouncing care for financial reasons

In French Guiana, during the past 12 months, 30.9% of surveyed persons renounced care whatever the type (dental, ophthalmology, general practitioner or other care) for financial reasons.

Results of the multivariate analysis showed that gender, perceived financial situation, perceived health and complementary insurance status were independent predictive factors of care renouncement for financial reasons (Table 3).

**Table 3:** Proportion and prevalence ratio for having renounced care for financial reasons

Variable		Having renounced care for financial reasons (%)	p*	Prevalence ratio**	p**
Gender (*N* = 2015)	Male	25.3	< 0.001	1	0.001
	Female	36.2		1.36 [1.14;1.63]	
Age (*N* = 2015)	15–24 years	23.6	0.010,		
	25–34 years	35.9			
	35–44 years	32.0			
	45–54 years	35.9			
	55–64 years	29.2			
	65–75 years	26.3			
Birth place (*N* = 2015)	Born in France	27.1	< 0.001		
	Foreign-born	37.7			
Educational level (*N* = 1994)	None	36.8	0.002		
	Below High school graduation	27.4			
	High school graduation	30.2			
	first college degree	30.0			
	Equal or above to a bachelor degree	23.1			
Language most often spoken (*N* = 2015)	French	26.9	< 0.001		
	Creole	38.6			
	Portuguese	45.4			
	English	31.9			
	Nengue tongo	26.1			
	Amerindians languages	33.8			
	Others	19.0			
Perceived Financial Status (*N* = 2000)	You are really comfortable	15.6	< 0.001	1	0.000.
	Your are comfortable	21.7		1.36 [0.96;1.93]	
	It is tight	32.2		1.96 [1.37;2.81]	
	It’s really difficult	49.1		2.78[1.99;3.89]	
	You do not make ends meet without making debts	55.4		2.90[1.98;4.25]	
Professional Status (*N* = 2014)	Employed	29.4	< 0.001		
	Student	19.4			
	Unemployed	39.1			
	Retired	21.4			
	Other inactive	42.5			
Living conditions (*N* = 2014)	Having water at home	29.7	0,020,0,020,		
	No water at home	41.3			
Residence location (*N* = 2015)	Coastal	33.1	0.137		
	Savanas	25.4			
	Western	27.6			
	Eastern	26.5			
Living alone (*N* = 2007)	Yes	30.6	0.369		
	No	34.1			
Having a religion (*N* = 2007)	Yes	33.9	0.001		
	No	25.1			
Perceived health (*N* = 2003)	Very good	23.1	< 0.001	1	0.0002
	Good	27.9		1.06 [0.82;1.37]	
	Fairly good	34.9		1.17 [0.90;1.51]	
	Poor / Very poor preceived health	63.1		1.71 [1.27;2.29]	
Having complementary health insurance (*N* = 1991)	Yes	28.8	< 0.001	1	0.000.
	No	40.1		1.44[1.20;1.74]	

^*^Pearson’s chi-square test in bivariate analysis

^**^Final multivariate model

The variable “Religion” and “Housing condition” were not included in the multivariate model because they describe various situations and profiles that did not allow interpretation

Living alone and residence location (coastal, Western, Eastern, Savannas) were not statistically associated with renouncing care for financial reasons. When distinguishing between types of care, 19% of persons declared renouncing dental care (19%), 14% renounced optical devices, and 12% renounced a consultation with a doctor (general practitioner: 8%, specialist: 5%).

### Non financial motives for renouncing care

Regarding the non-financial motives for renouncing care, 9% renounced because of the distance of the health care professional (7% in mainland France) and 23% (vs 22% in mainland France) because the delay to obtain an appointment was too long. In French Guiana, however, there were a greater proportion of persons renouncing for transport difficulties: 12% vs 8% in other French overseas territories and 6% in mainland France. This motive was often cited by younger age groups: 15% of 15–30 years (7%), 12% of the 31–45 years (5%), 7% among persons aged over 45 years (6%).

Among those having declared renouncing for financial reasons in the past year, 37.5% also declared renouncing because appointment delays were too long.

When pooling all the different motives linked to time, 24% of the surveyed population declared having renounced to care for time-related motives including appointment delays, waiting time, and time in general. The independent predictors for time-related renouncing were different than those for renouncing care for financial reasons: a higher education level and a poor perceived health were independently associated with time-related renouncement; retired persons and students were found to renounce care less frequently than persons with a job (Table 4).

**Table 4:** Percent and prevalence ratio for having renounced care for time-related reasons

Variables		Have renounced for time-related reasons (%)	p*	Prevalence ratio**	p**
Gender (*N* = 2012)	Male	21.4	0.031		
	Female	26.5			
Age (*N* = 2012)	15–24 years	20.5	0.011		
	25–34 years	26.9			
	35–44 years	29.3			
	45–54 years	25.4			
	55–64 years	15.9			
	65–75 years	16.3			
Birth place (*N* = 2012)	Born in France	25.2	0.205		
	Foreign-born	21.9			
Educational level (*N* = 1991)	None	19.9	< 0.001	1	0.0015
	Below High school graduation	23.06		1.21 [0.90;1.62]	
	High school graduation	31.2		1.64 [1.23;2.18]	
	first college degree	26.8		1.36 [0.94;1.96]	
	Equal or above to a bachelor degree	33.7		1.70 [1.25;2.32]	
Perceived Financial Status (*N* = 1997)	You are really comfortable	17.7	0.031		
	Your are comfortable	24.01			
	It is tight	25.6			
	It’s really difficult	23.8			
	You do not make ends meet without making debts	35.5			
Language most often spoken (N = 2012)	French	26.5	0.239		
	Creole	19.3			
	Portuguese	28.7			
	English	20.4			
	Nengue tongo	19.2			
	Amerindians languages	21.5			
	Others	20.4			
Professional Status (N = 2012)	Employed	27.9	0.013	1	0.02
	Student	18.8		0.73 [0.53;1.00]	
	Unemployed	23.7		0.96 [0.72;1.29]	
	Retired	13.8		0.50 [0.31;0.80]	
	Other inactive	19.7		0.80 [0.51;1.26]	
Living conditions (*N* = 2011)	Having water at home	24.6	0.238		
	No water at home	19.1			
Residence location (*N* = 2012)	Coastal	24.7	0.539		
	Savanas	20.8			
	Western	25.2			
	Eastern	17.7			
Having a religion (*N* = 2005)	Yes	23.9	0.834		
	No	24.4			
Perceived health (*N* = 2000)	Very good	19.9	0.067	1	0.0043
	Good	24		1.19 [0.89;1.58]	
	Fairly good	25.4		1.35 [0.99;1.84]	
	Poor / Very poor	34.3		2.06 [1.37;3.12]	
Having complementary health insurance (*N* = 1989)	Yes	23.9	0.791		
	No	24.8			

*Pearson’s chi-square test in bivariate analysis

**Final multivariate model

As detailed in Table 4, the significant predictors of renouncing care for time-related reasons were educational level, professional status and health perception.

**Fig. 2:**
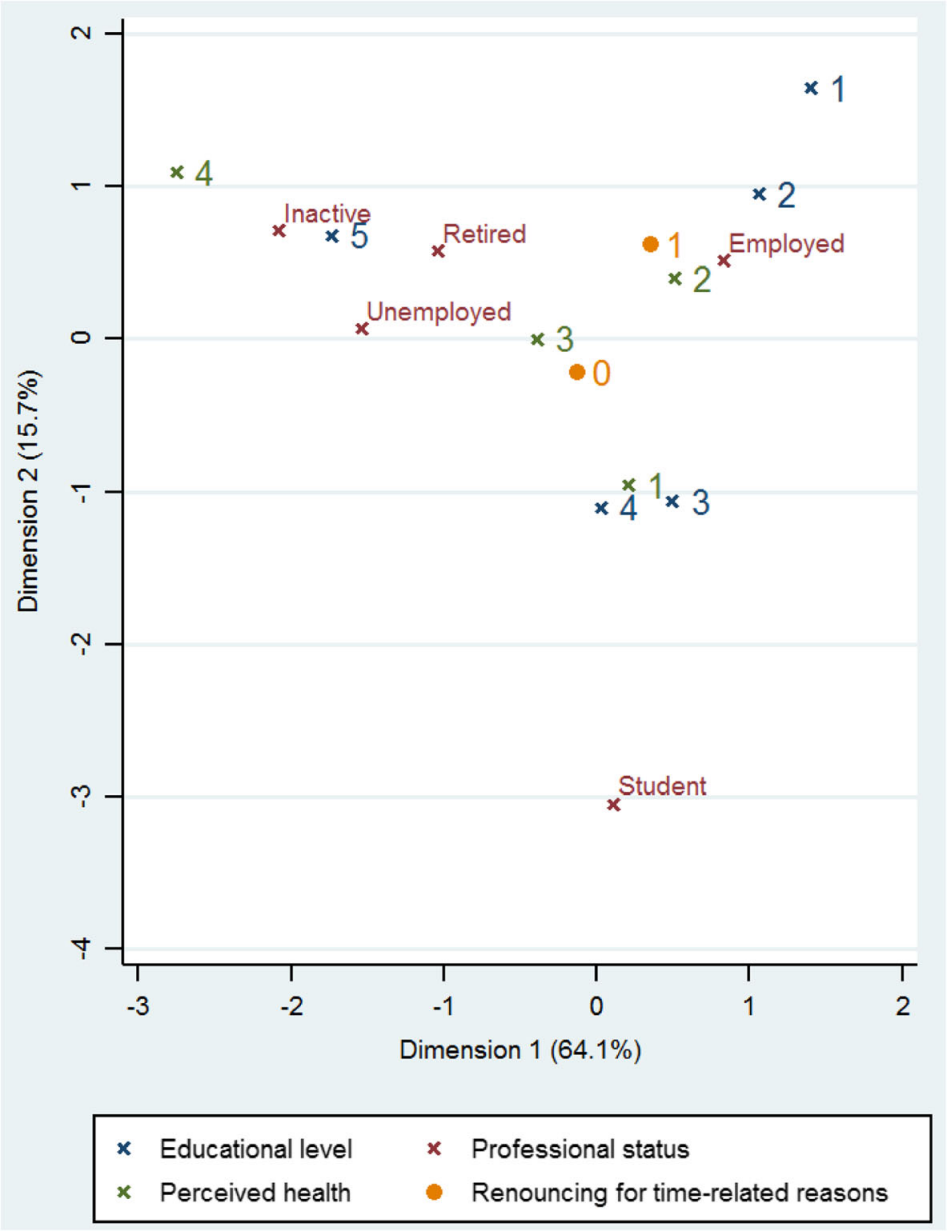
Multiple Correspondence Analysis: renouncing care for time-related reasons. Legend: Educational level:1: Equal or above to a bachelor degree; 2: First college degree or ordinary college degree; 3: High school graduation; 4: Below High school graduation; 5: None; Perceived health:1: Very good; 2: Good; 3: Fairly good; 4: Poor/Very poor. Renouncing care for time-related reasons: 0 = No; 1 = Yes

Although the variables independently associated with renouncing care for time-related reasons were not collinear, they were linked and significant interactions were found. To simplify the interpretation of results, multiple correspondence analysis was performed to search for a particular profile of persons who renounced for time-related motives. Figure 2 breaks represents the variable projections on two major axes of variability. Variable points that are clustered in the two dimensional space tend to vary in the same direction and suggest homogeneous groups. These descriptive results highlighted a subgroup of persons that were professionally active, with an educational level equal or higher than 2 years of university, and having a good perception of their own health status as more susceptible to renounce care for time-related reasons (Fig. 2).

## Discussion

The sample mostly included French-speaking persons and did not include persons who do not speak Creole or French who may be the most vulnerable population. In addition, the survey sample underrepresented persons living in the remote areas of French Guiana (12% vs 30% at the last census) who are far from specialized care and centers allowing access to their rights, and are often socially vulnerable. The questionnaire was a standard tool used in different parts of France and did not aim for the specifics of French Guiana, therefore we could not explore particular vulnerable groups. Despite this, renouncing for financial reasons represented a public health problem in French Guiana. Renouncing for financial reasons is an indicator of social inequalities of health which are a major target of the 2016 health law.

French Guiana, a French territory, has a social system that aims towards universal coverage. However, the structural development delays, the problems of low health professional demographics (55/100000 general physicians versus 104/100000 in mainland France [http://drees.solidarites-sante.gouv.fr/IMG/apps/statiss/default.htm]), the weight of precariousness on the severity of diseases already reflect the contrast between French Guiana and mainland France. For French Guiana, the health system often uses comparisons with mainland France as a national benchmarking reference to evaluate the performance, and often to point at perceived national inequities in the allocation of resources in the republic. The question of renouncing care reveals another subjective side to the state of health care. Thus, in French Guiana, during the past 12 months, 30.9% of surveyed persons had renounced care for financial reasons (12.2% a consultation, 19% dental care, and 13.8% optical care). The level of renouncement to a medical consultation was higher than in mainland France (12.2% vs 7.6%). The level of renouncement to optical care was significantly higher than in mainland France (14% vs 12%).The level of renouncement for dental care was similar than in mainland France (19% vs 17%).

After multivariate analysis, the variables remaining independently associated with renouncing care for financial reasons were classical [23, 28]: women were more likely to renounce, those with a poor perception of their health, those with the lowest incomes, those without a complementary health insurance; the multivariate analysis also showed that those who were retired or students were less likely to renounce for financial reasons. Women may renounce more for individual reasons (often a poorer perception of their health) but in addition to individual reasons, there may be important contextual and cultural reasons, such as the constraints of school schedules, family roles, which may not be necessarily taken into account in the health services’ organization. It was counter intuitive and thus noteworthy that residence location was not associated with any significant differences regarding renouncing care for financial reasons. However, the underrepresentation of certain remote areas may be an explanation.

The concern for equity in access to health and healthcare, and the evaluation of social programs make renouncing for financial reasons an important indicator. Moreover, renouncing care or not seeking care for non-financial reasons may also have dramatic health consequences. The present study showed that 22.9% of persons declared renouncing care because delays for an appointment were too long. Among those who had declared renouncing for financial reasons, 37.5% also declared renouncing because the delays for an appointment were too long. When considering all motives for renouncing (appointment delays, waiting time, time in general), 24% of the surveyed population declared renouncing for reasons pertaining to discrepancy between delays to get care and patient’s available time. In addition transport problems were also a motive for renouncing care, and more so in French Guiana (12%) relative to mainland France (5.8%).

Since a variety of personal, interpersonal and situational factors influence health decisions, inducing modifying health behavior may be attempted at various points in the decision process: minimizing barriers, maximizing convenience, providing effective cues to action may increase acceptance of health programs [13]. However, despite these attempts, some will remain unresponsive to the efforts of the health system. For these persons efforts will need to be focused directly on their beliefs or their behavior. Educational programs designed to increase life skills and the acceptance of the beliefs as well as the adoption of preventive health behavior should be directed primarily to the most vulnerable groups. However, a process of self-selection often leads these target groups to not to consume scientific and health information transmitted through the mass media, which have not been particularly effective in changing health beliefs and behavior. Personal influence in face-to-face contacts may be more effective in educating members of the affected groups. It is not clear whether school health programs have a lasting effect on the acquisition of health beliefs and behaviors. Research suggests the potential value of emotionally arousing factors in education and the importance of the conditions affecting the effects of emotionally arousing messages on attitude and behavior change [13]. Community action is more efficient, in health education and developing life skills, but also in empowering the population and enhancing the participation of patients in the health care system.

The present results suggest different action possibilities to avoid health care delays and their potential complications. Renouncing for reasons of excessive delays affected different social strata. This was an unsurprising fact given the relative lack of health professionals in French Guiana [16]. The association of renouncement for reasons linked to time with professional status and educational level suggested that among those who are employed, taking half a day off to consult is often problematic. This raises questions. Indeed, with internet, as it is the case for hotels, flights and a number of products in real time, it would be technically easy to know the availability of all health professionals. Regarding health, with a few exceptions, it remains difficult to guess where one would see a doctor with the least possible delay in order to conciliate medical availability and patient availability. This consumer patient vision clashes with the situation of the family practitioner in the French system, but it would potentially allow reducing a non-negligible proportion of renouncing care across all socio economic strata. However, this would presumably not reduce the social inequalities of health and would mostly concern the urban areas which have the most health professionals.

As for renouncing for financial reasons, as elsewhere in France, the improvement in access to complementary health insurance was associated with less renouncing for financial reasons. It is tempting to impute this to the fact that complementary health insurance allows persons to access care. However, it is not possible to exclude a bias where those who undertake the administrative procedures to access complementary insurance are also the most persistent persons who will also persist to access care. Concerning the fuzzy area of non demand (not requesting health insurance, not requesting care), which also leads to health care delays and potentially more advanced diseases, local social development actions have been recommended for the concerned populations [29].

In the USA, patient navigators are used in oncology, HIV disease and other chronic pathologies [30]. These approaches are officially recommended notably by the Centers for Disease Control and Prevention (CDC) and in France by the “Haut Conseil de la Santé Publique” which refers to mediators of the health care trajectory*.* This question is regularly debated in French Guiana but remains fragmented, disease-specific, poorly funded, with no clear professional status for mediators. The 2016 health law has reemphasized the importance of health inequalities and has recommended health mediators to reduce social inequalities of health [31]. Measures are now taken to stabilize health care insurance, and to precisely define the required curriculum of health mediators. However, despite the general aim of stabilizing health insurance, for migrants with short term residence permits the new reforms may make matters much worse by further accelerating the need to apply for health insurance, every time the residence status changes. This is therefore unlikely to result in less renouncement to care in this vulnerable group.

## Conclusion

There are marked health inequalities in French Guiana. There is a social gradient of risk factors, of health knowledge and preoccupations, of the capacity to understand and navigate the health system, and thus for the accessibility of the health system. There are avenues for improvement of health for the most vulnerable: promote health, act on risk factors, and facilitate the readability and accessibility of the health system. Not soliciting the health care system and renouncing healthcare are more frequent than in mainland France, the national benchmark for performance of French territories. Renouncing for lack of time was an important motive for renouncing, which is aggravated by the insufficient number of health professionals, but may benefit from organizational solutions. The proposed solution to enhance the link with the poorest populations raises the question of the required organization and adequate scaling up of health mediators. Strategic choices will be needed to allocate scarce resources to the health problems causing the highest burden of disease.

## Abbreviations

GDP per capita: Gross Domestic Product per capita
HIV: Human Immunodeficiency Virus
VIF: Variance inflation factors
